# Improving Biodegradable Mg-Zn(-Ca) Alloys by Surface Treatment via Plasma Electrolytic Oxidation

**DOI:** 10.3390/ma18040747

**Published:** 2025-02-08

**Authors:** Jakub Vertaľ, Daniel Kajánek, Jiří Kubásek, Peter Minárik

**Affiliations:** 1 Charles University, Department of Physics of Materials, Ke Karlovu 5, 121 16 Prague, Czech Republic; kubovertal@gmail.com; 2 University of Žilina, Research Centre, Univerzitná 8215/1, 01026 Žilina, Slovakia; daniel.kajanek@uniza.sk; 3 University of Chemistry and Technology, Department of Metals and Corrosion Engineering, Technická 5, 166 28 Prague, Czech Republic; jiri.kubasek@vscht.cz

**Keywords:** magnesium, plasma electrolytic oxidation, microstructure, corrosion

## Abstract

This study investigated the influence of plasma electrolytic oxidation (PEO) preparation time on the degradation resistance of Mg-1Zn (Z1) and Mg-1Zn-0.4Ca (ZX10) alloys, with comparisons to pure Mg and commercial Mg-4Y-3RE-0.4Zr (WE43). PEO layers were formed with varying preparation times (5, 10, and 15 min) and analyzed for microstructure, morphology, and corrosion resistance. The results indicated that PEO layers with a 10 min preparation time had the most homogeneous structure and optimal corrosion resistance. Prolonged PEO preparation times increased pore density, crack formation, and layer thickness while also promoting layer degradation during extended immersion in 0.9% NaCl corrosive media. The dissolution of phosphates from PEO layers contributes to the formation of a protective corrosion layer, enhancing long-term resistance. These findings demonstrate that low-alloyed, biocompatible Mg-Zn(-Ca) alloys can achieve corrosion resistance comparable to high-performance WE43 alloys through appropriate surface treatment.

## 1. Introduction

The development of magnesium (Mg) alloys for medical applications has been the focus of intensive research in recent years, yielding significant advancements, including the commercialization of the first biodegradable implants [1,2]. However, a critical challenge limiting their application, particularly in the production of larger biodegradable medical devices such as bone fixation systems, is the combination of inadequate corrosion resistance during the initial implantation period and the unpredictable nature of corrosion attack. To mitigate these limitations, implants are typically designed with increased thickness to prevent mechanical failure during the healing process.

One promising approach to address this issue is the formation of a surface layer that provides high corrosion resistance while remaining biodegradable. This layer is intended to delay the onset of substantial corrosion until the surrounding tissue has healed, rendering the material’s mechanical strength less critical [3].

In this study, special attention was devoted to plasma electrolytic oxidation (PEO), a surface treatment that generates a ceramic-like oxide coating on the material’s surface. This layer, typically with a thickness of up to tens of microns, exhibits excellent corrosion resistance and high strength, with strong adhesion to the substrate due to the chemical bonding between the oxide layer and the metal [4]. The PEO process is similar to anodizing, but operates at higher voltages—up to 600 V—resulting in dielectric breakdown and plasma discharges on the sample surface. During the process, molten oxide is expelled through channels in the material, solidifying it into a hard, porous layer upon contact with the cold electrolyte [5]. However, the PEO layer’s porous and cracked structure is a key drawback, as these defects compromise its corrosion resistance. The composition of the electrolytic solution, which may include aluminate-, phosphate-, or silicate-based components, also influences the layer’s properties [6]. This surface treatment does not depend on the sample shape, is affordable, and has a scalability that depends mainly on the capacity of the power source. Therefore, it is very interesting in terms of samples of complex shapes, such as fixation implants.

The composition of the PEO layer strongly depends on the composition of the substrate and the electrolyte [7]. Therefore, there are strong limitations for biodegradable applications, which pose strict requirements for both composition and biocompatibility. In this regard, the phosphate electrolyte is a very good choice for biodegradable magnesium alloys and was repeatedly used in different in vivo [8,9] and in vitro studies [10,11,12,13] with very promising results.

Among the Mg alloys frequently employed in biomedical applications, Mg-Zn-Ca alloys stand out due to the biocompatibility of both zinc and calcium [14,15]. Zinc enhances the alloy’s mechanical properties through solid solution strengthening and grain boundary strengthening [16], while calcium contributes to improved strength and serves as a vital component of bone and teeth, playing essential roles in blood clotting and nerve function [17]. A representative example of such alloys is Mg-1Zn-0.4Ca (ZX10, wt.%), which has been extensively studied for biodegradable applications across various microstructural states, including ultrafine-grained conditions [18]. ZX10 exhibits favorable mechanical properties, high strength, and a controlled, homogeneous degradation process with minimal hydrogen evolution [17,19]. The PEO formation on ZX00 (<1Zn, <1Ca, wt%) [9] and ZX20 (2.1Zn, 0.6Ca, wt%) [13] already showed a significant increase in the corrosion resistance in this class of magnesium alloys. However, the first study focused primarily on in vivo degradation with only limited characterization of the PEO layer, and the second one focused on the incorporation of Mn_3_O_4_ nanoparticles into the PEO layer. Therefore, a comprehensive characterization of PEO layer formation and its effect on the corrosion resistance of the well-known ZX10 alloy is still missing.

This work aimed to investigate the influence of PEO preparation time on the degradation resistance of Mg-1Zn (Z1) and Mg-1Zn-0.4Ca (ZX10) alloys. The comparison of Z1 and ZX10 alloys serves to elucidate the role of calcium during PEO formation and resulting corrosion resistance in the case of this class of magnesium alloys. For comparative analysis, pure Mg and commercial Mg-4Y-3RE-0.4Zr (WE43) alloy were subjected to similar preparation and testing conditions.

## 2. Materials and Methods

### 2.1. Materials and Preparation of PEO

This study investigated three magnesium alloys—Mg-1Zn (Z1), Mg-1Zn-0.4Ca (ZX10), and Mg-4Y-3RE-0.4Zr (WE43)—and pure magnesium (Mg, 99.96%). The Z1 and ZX10 alloys were fabricated through conventional casting, and their chemical composition was analyzed using energy-dispersive X-ray spectroscopy (EDS). The WE43 alloy and pure Mg were obtained commercially in extruded and cast forms, respectively. Prior to experimentation, all alloys underwent annealing for 16 h at 530 °C in an argon atmosphere to homogenize their chemical composition and dissolve secondary phases.

The PEO process was performed with the samples serving as the anode and a stainless-steel sheet acting as the cathode using a N8762A DC power source (Keysight, Santa Rosa, CA, USA). To ensure homogeneity of the electrolyte, a rotating stirrer was employed. The electrolyte consisted of 12 g/L Na_3_PO_4_·12H_2_O and 1 g/L KOH, and a constant current density of 50 mA/cm^2^ was applied to the anode. In our previous study, these preparation parameters were found to be the most beneficial for PEO thickness and compactness [20]. Samples with dimensions of 15 × 15 × 5 mm^3^ were exposed to the PEO process for three different durations—5, 10, and 15 min—to optimize coating morphology and corrosion resistance. Prior to PEO, all samples were mechanically ground and polished using a 3 µm diamond suspension, followed by electro-polishing with a Lectropol device (Struers, Ballerup, Denmark) and Struers AC2 solution.

### 2.2. Microstructure

The microstructure of the alloys was examined using optical microscopy. Sample preparation for microstructural analysis followed the same protocol as for pre-PEO treatment. Samples were etched with a solution comprising 10 mL acetic acid, 4.2 g picric acid, 10 mL H_2_O, and 70 mL ethanol. Microstructural observations were conducted using a LSM 700 optical microscope (ZEISS, Oberkochen, Germany).

Morphological, cross-sectional, and compositional analyses of the PEO layers were carried out using a APREO 2 scanning electron microscope (SEM; ThermoFischer, Waltham, MA, USA) equipped with energy-dispersive X-ray spectroscopy (EDS; EDAX, Pleasanton, CA, USA). Samples for cross-sectional observations were embedded in conductive epoxy resin, ground, and polished with a 50 nm alumina suspension.

### 2.3. Corrosion

Corrosion resistance was evaluated using electrochemical impedance spectroscopy (EIS) with a VSP-300 potentiostat (Biologic, Seyssinet-Pariset, France) in a three-electrode configuration. The frequency range was set between 100 kHz and 10 mHz, with frequency steps of 10 per decade and a potential amplitude of 10 mV. A 0.9% NaCl solution at room temperature served as the corrosive medium. Samples were exposed for up to 168 h to monitor degradation processes. Additionally, the corrosion resistance of PEO-free samples was measured after 2 h of exposure for each alloy.

## 3. Results

### 3.1. Initial Microstructure

**Figure 1:**
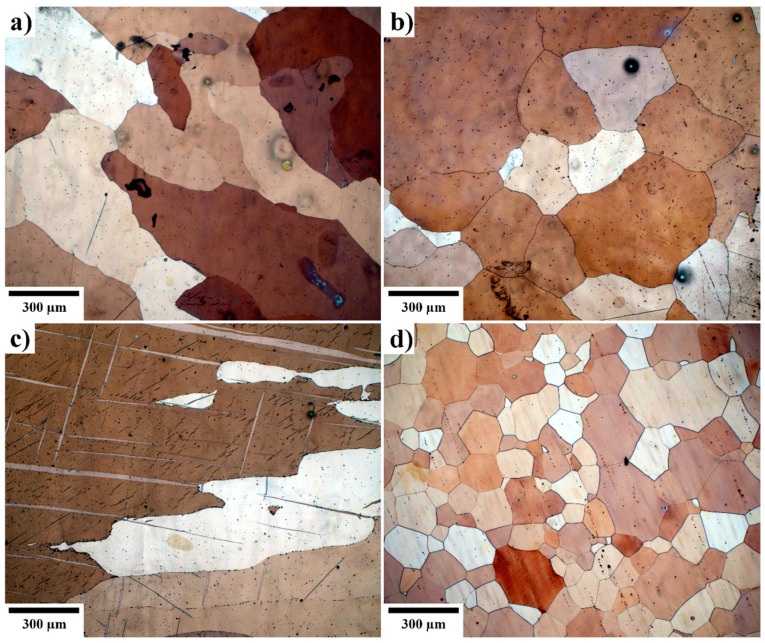
Optical micrographs of the initial microstructure before PEO preparation. (**a**) Z1, (**b**) ZX10, (**c**) pure Mg, and (**d**) WE43.

The initial microstructure of the investigated alloys was analyzed using optical microscopy on etched surfaces. As shown in Figure 1, no secondary phases comprising alloying elements are observed. This absence is attributed to the solution annealing process performed during production, which ensured the dissolution of alloying elements into the material matrix.

The microstructures of the ZX10 and Z1 alloys, as well as pure magnesium, were characterized by irregularly shaped, large grains. The grains in the ZX10 and Z1 alloys were comparable in both shape and size. The average grain size evaluated via the linear interception method was ~0.49 mm and ~0.32 mm for Z1 and ZX10, respectively. In contrast, the grains in pure magnesium were significantly larger, with an average grain size of ~1.35 mm. The WE43 alloy represented an exception, as it was supplied in an as-extruded condition. Following homogenization treatment, the WE43 alloy exhibited a microstructure composed of polyhedral grains. Notably, the WE43 alloy had the smallest average grains among the tested materials, approximately ~0.15 mm, making it distinct from the other alloys.

### 3.2. PEO Layers

As anticipated, the plasma electrolytic oxidation (PEO) layers of all samples exhibited a porous structure. These pores are formed due to the rapid cooling of molten oxide when it contacts the cold electrolyte. Simultaneously, this interaction generates an accumulation of voltage, resulting in the formation of microcracks within the layer structure.

**Figure 2:**
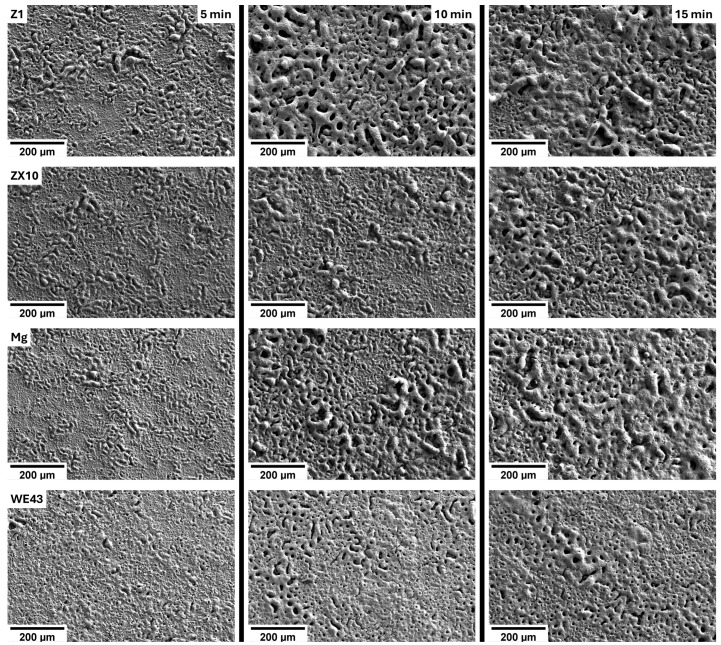
The topography of the PEO layers formed on the investigated materials after three preparation times.

Figure 2 presents the topography of the PEO layers developed on the studied alloys for three different preparation times. After 5 min of preparation, the PEO layers were predominantly characterized by a high surface density of small pores (<1 µm). Notably, the Z1 alloy was the only sample to exhibit larger pores (>10 µm) surrounded by thicker ridges in the PEO layer. Cross-sectional measurements indicated an average PEO layer thickness of approximately 12 µm, with the exception of the WE43 alloy, which exhibited a thinner layer of ~7 µm (see Table 1). These measurements were conducted in pore-free regions of the layers.

Increasing the preparation time to 10 min resulted in a marked rise in the surface density of large pores, particularly in the Z1 and Mg samples. In the Z1 alloy, large pores became the dominant feature, covering most of the surface. In other samples, large pores formed clusters interspersed with regions of smaller pores. The WE43 alloy displayed the lowest surface density of large pores and did not develop pronounced ridges around them, as observed in other materials. A higher surface density of large pores was found in the ZX10 alloy, followed by the Mg sample, where these pores covered more than three-quarters of the surface area. This extended preparation time also increased the thickness of the PEO layers. As shown in Table 1, the Z1 and Mg samples exhibited thicker layers (~18 µm) compared to the ZX10 and WE43 alloys (~14 µm). The greater thickness in Z1 and Mg samples is attributable to the higher surface density of large pores and more prominent ridges.

Further, increasing the preparation time to 15 min did not induce substantial qualitative changes in the topography of the PEO layers for individual samples. The Z1 and Mg samples continued to exhibit the highest surface density of large pores, which, along with high ridges, covered nearly the entire surface. The ZX10 alloy displayed a modest increase in large pore density, with its surface appearing similar to that of the Mg sample after 10 min of preparation. Notably, the PEO layer of the WE43 alloy remained largely unchanged even after 15 min, with the surface maintaining a highly homogeneous distribution of predominantly small pores. Occasional clusters of large pores were observed, but these were not surrounded by the prominent ridges seen in the other materials. The thickness of the PEO layers increased further with the extended preparation time, reaching comparable levels (~23 µm) across all alloys, except for the WE43 alloy, which developed the thickest layer at approximately 27 µm.

**Figure 3:**
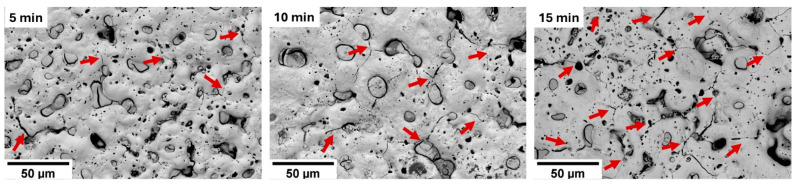
Development of the cracks, marked by red arrows, in the PEO layer with increasing preparation time for the Z1 alloy (BSE signal).

Cracks were observed in the PEO layers of all investigated samples. Figure 3 provides a detailed depiction of crack development in the Z1 alloy. The formation and progression of cracks showed a systematic relationship with the preparation time, both in terms of their quantity and thickness. After 5 min of preparation, cracks were predominantly localized near larger pores and appeared unconnected. However, as the preparation time increased, these cracks began to interconnect, and their thickness expanded progressively. By 15 min, the PEO layer exhibited a relatively dense network of interconnected cracks, as illustrated in Figure 3. This trend of crack development, characterized by increasing connectivity and thickness with longer preparation times, was consistently observed across all studied materials.

**Figure 4:**
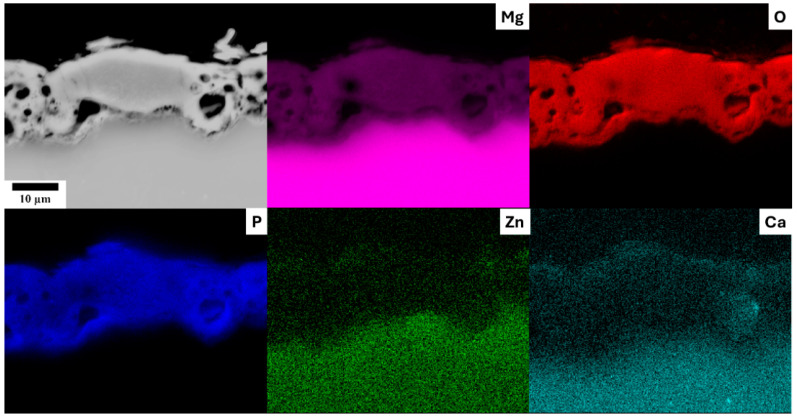
Cross section of the PEO layer formed on the ZX10-10 min sample with the chemical composition analysis.

The investigation of PEO cross sections revealed significant heterogeneity within the layers. Large pores frequently extended almost to the substrate surface, contributing to the formation of cavities, as demonstrated in Figure 4 for the ZX10 alloy prepared with a 10 min treatment time. Notably, layers produced with a 10 min preparation time were systematically the most compact across all the investigated alloys. Figure 4 also presents EDS elemental maps of the PEO layer cross section, indicating that the layer primarily consists of magnesium, oxygen, and phosphorus. This is a typical composition of PEO formed in a phosphate-rich solution, resulting in the formation of Mg_3_(PO_4_)_2_ and MgO [7]. Their formation follows the equations:

Mg → Mg^2+^ + 2e^−^ (1)

Mg^2+^ +2OH^−^ → Mg(OH)_2_ (2)

Mg(OH)_2_ → MgO + H_2_O (3)

Mg^2+^ + 2PO_4_^3−^ → Mg_3_(PO_4_)_2_ (4)

in which (1) represents the magnesium dissolution reaction, (2) and (3) represent the formation process of MgO, and (4) represents the formation of Mg_3_(PO_4_)_2_.

Small amounts of zinc and calcium were detected, indicating that their concentrations were considerably lower compared to those in the substrate. Note that this relative decrease in alloying elements concentration is primarily due to the PEO layer enrichment by oxygen and phosphorus. The amount of zinc and calcium was comparable to the matrix composition when only ratios between magnesium and alloying elements were considered. These findings were consistent across all studied alloys, irrespective of their composition, as shown in Table 2. Interestingly, in the ZX10 alloy, a small PEO layer enrichment by calcium was observed, indicating a possible formation of calcium oxide or calcium phosphate. However, no direct observation of such phases was achieved.

### 3.3. Corrosion Resistance

**Figure 5:**
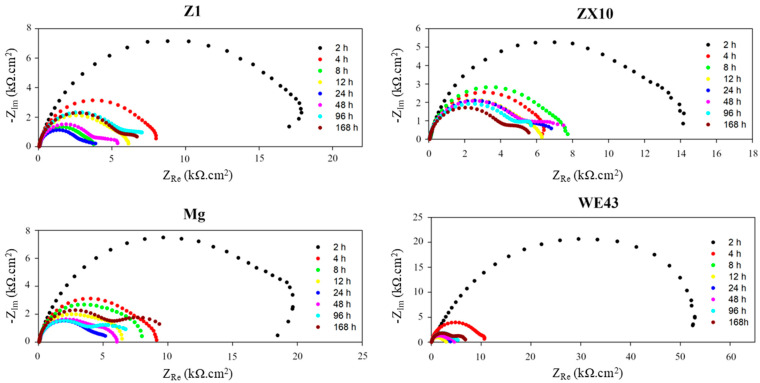
Representative Nyquist plots of the investigated 10 min samples.

**Figure 6:**
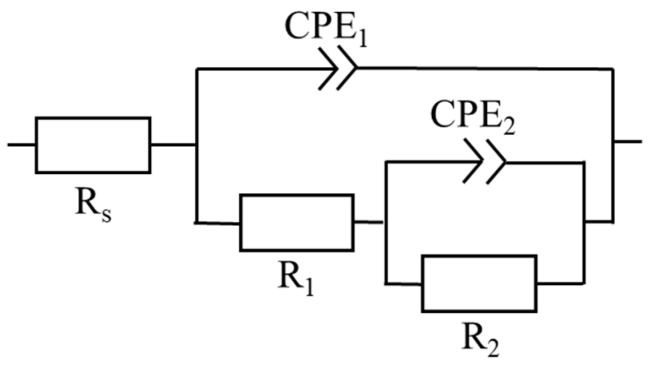
Equivalent circuit used for the EIS data analysis.

The protective performance of the PEO layers was evaluated in a 0.9% NaCl solution over a one-week exposure period. The corrosion resistance of the alloys with PEO coatings was monitored periodically by electrochemical impedance spectroscopy (EIS) at intervals of 2 h, 4 h, 8 h, 12 h, 24 h, 48 h, and 168 h. The representative Nyquist plots are shown in Figure 5 for all 10 min samples. The experimental data were fitted using the equivalent circuit shown in Figure 6, which most effectively represents the physical phenomena occurring at the PEO layer. Two capacitive loops represent the occurrence of areas with different electrochemical behavior, usually referring to the coating/corrosion product layer (first loop) and the existence of a sub-layer or the presence of a double layer on a surface (second loop). In this model, the parameter R_S_ denotes the solution resistance, while R_1_ and R_2_ represent the charge transfer resistance through distinct regions of the layer. A detailed description of the physical meaning of the individual parameters of the equivalent circuit is given elsewhere [21,22]. Nevertheless, from the corrosion resistance point of view, the most important parameter is the total impedance R_sum_ = R_1_ + R_2_, which represents the total corrosion resistance of the material. Note that during certain measurements (see Figure 5), the Nyquist plot displayed only a single arc. In these instances, the R_2_ and CPE_2_ elements were excluded from the equivalent circuit.

**Figure 7:**
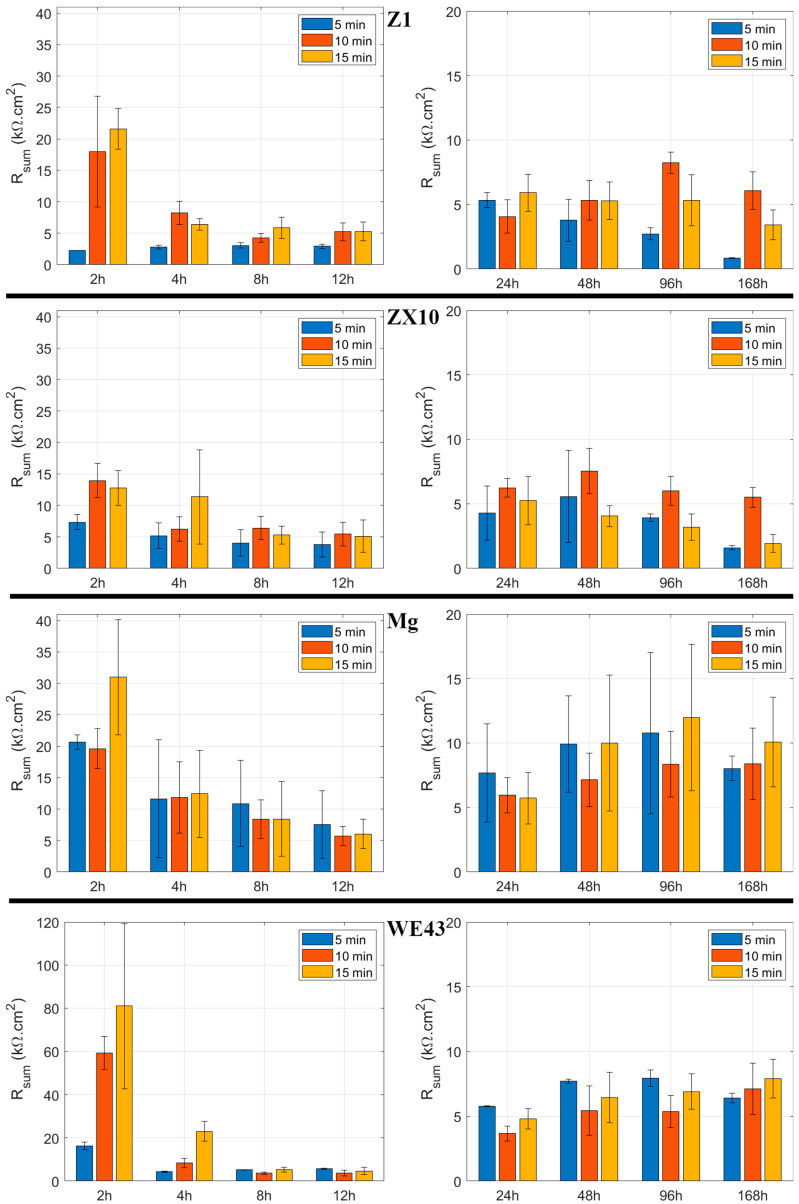
Graphical representation of R_sum_ values of the PEO layers with different preparation times.

The polarization resistance of the uncoated samples measured after 2 h of exposure was (3.6 ± 0.2) kΩ·cm^2^ for Z1, (3.6 ± 0.1) kΩ·cm^2^ for ZX10, (0.82 ± 0.02) kΩ·cm^2^ for Mg, and (4.7 ± 0.2) kΩ·cm^2^ for WE43. Clearly, the formation of the PEO layer significantly affected the corrosion resistance of the materials, as shown in Figure 7. Generally, samples with PEO layers formed for longer durations (10 and 15 min) exhibited higher corrosion resistance than those formed for 5 min. The polarization resistance after 2 h of exposure was approximately 20 kΩ·cm^2^ for the Z1 alloy (10 and 15 min samples), whereas it was only around 14 kΩ·cm^2^ for the ZX10 alloy (10 and 15 min samples). For the Mg sample, the highest resistance was observed in the Mg-15 min sample (~30 kΩ·cm^2^), and for the WE43 alloy, the highest value was approximately 80 kΩ·cm^2^ for the WE43-15 min sample. However, due to considerable data scatter, the WE43-15 min sample can be considered statistically similar to the WE43-10 min sample, which had a resistance of ~60 kΩ·cm^2^. As the exposure time increased, all samples exhibited a continuous decrease in corrosion resistance, reaching approximately 5 kΩ·cm^2^ after 24 h. The only exception was the Z1-5 min sample, which initially showed a relative decrease in polarization resistance after PEO formation (~2.3 kΩ·cm^2^), followed by a gradual increase in resistance over time, reaching ~5.3 kΩ·cm^2^ after 24 h.

After 24 h of immersion, the corrosion resistance of nearly all samples began to increase, as shown in Figure 7. The notable exceptions were the Z1-5 min and ZX10-15 min samples, which displayed a continuous decrease in resistance with prolonged immersion time. After 168 h of immersion, the highest polarization resistance among the Mg-Zn(-Ca) samples was observed in the Z1-10 min ((6.1 ± 1.5) kΩ·cm^2^) and ZX10-10 min ((5.5 ± 0.8) kΩ·cm^2^) samples. The Mg and WE43 samples exhibited resistances of (8.4 ± 2.8) kΩ·cm^2^ and (7.1 ± 2.0) kΩ·cm^2^, respectively, across all PEO preparation times. Although there were relatively significant differences in the average corrosion resistance values, the high data scatter after one week of immersion observed in all samples indicates that these results are statistically comparable within the measurement error.

**Figure 8:**
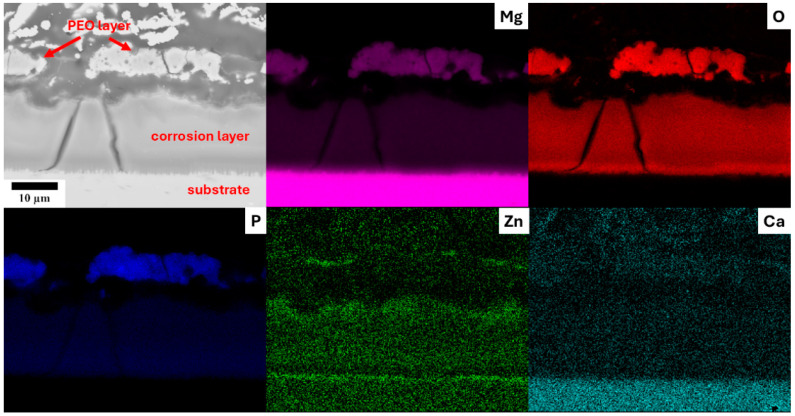
Cross section of the PEO layer formed on the ZX10-10 min sample after 168 h of immersion in 0.9% NaCl solution, including the chemical composition analysis.

The examination of the cross section through the surface layer after 168 h of immersion revealed significant dissolution of the PEO layer during the degradation process in 0.9% M NaCl solution, with the formation of a compact corrosion layer beneath it. This phenomenon was observed for all samples investigated. Figure 8 illustrates this degradation in the ZX10-10 min sample, where the average thickness of the PEO layer decreased to approximately 8 µm and was severely shattered. EDS elemental mapping indicated that phosphorus from the dissolved PEO layer was incorporated into the corrosion layer, which also contained zinc from the substrate material. Note that phosphorous was not present in the corrosion solution, and the PEO layer was the only possible source. Additionally, zinc was found to segregate at the interface between the substrate and the corrosion layer. In contrast, no calcium enrichment was detected in the corrosion layer. Elemental mapping of the Z1 alloy yielded similar results (excluding calcium), while in the case of the WE43 alloy, an enrichment of the corrosion layer with rare earth elements was observed.

## 4. Discussion

The formation of PEO is associated with the flow of molten oxide inside the electric discharges, which forms on the surface during the preparation. The molten oxide gradually lies over the previous layers, and the thickness of the coating grows. The formation of pores and cracks inside the PEO is inseparable from this process, and a lot of work has been done to minimize their size and density [23]. Most of the processing parameters (the solution composition and the current density) were chosen according to a previous study on commercial AZ31 [20], and the only free variable used in this study was the preparation time. The results showed that the gradual increase in PEO layer thickness with time was relatively comparable between the studied materials and was in the range of 1.4–1.7 µm/min. These values are significantly higher than the measured 0.9 µm/min for the polished AZ31 prepared with the same parameters [24]. Nevertheless, a growth rate of 0.5–1.6 µm/min is commonly observed for magnesium alloys [13,25], and the PEO thicknesses achieved in this study are comparable to other work [6,26]. For a very short preparation time (1 min), a growth rate of 3 µm/min can be achieved [10], but the further increase in thickness is evidently slowed by the formation of a non-conductive surface [12]. Therefore, the measured results are in accordance with the literature. In general, the direct comparison to other studies is problematic because of high variation in the processing parameters.

Figure 2 shows that the more alloyed materials (ZX10 and WE43) develop higher surface density of large pores later than the Mg and Z1 materials. In addition, the WE43 alloy, having the highest content of alloying elements, showed the lowest porosity even after 15 min of preparation. It was found that the decrease in porosity may be achieved by the increase in the surface defect density and topography prior to the PEO preparation [24]. However, this is not the reason for the smoother PEO surface of the WE43 alloy, as the grain size was on one hand lower than in the case of the other investigated materials, but on the other hand, it was significantly higher than the pore size and average distance between the pores. A small decrease in porosity with increasing content of alloying elements has already been reported [27], but the general reason was not revealed. In this study, the lower porosity was observed in the alloys with a higher content of alloying elements—WE43 and ZX10. This is clearly visible in Figure 2, particularly in the 10 and 15 min samples. The chemical analysis revealed that the PEO layer had a comparable ratio between the substrate alloying elements: Mg/Zn(/Ca) for Mg-1Zn(-0.4Ca) and Mg/Y/Nd for WE43. Therefore, the alloying elements were built into the oxide layer during the PEO process, which had some effect on discharges and consequently porosity. However, no particles rich in alloying elements were observed in this study. This statement also holds for the calcium in the ZX10 alloy, which was the only element observed with increased concentration in the PEO layer compared to the substrate. Note that the formation of calcium phosphates is convenient when an excess amount of calcium and phosphates is available during anodization [28]. However, no direct observation of this phase was achieved. It was shown that with increasing preparation time of the AZ-type alloys as a substrate, a MgAl_2_O_4_ spinel formed in the inner PEO layer [29,30]. Nevertheless, more alloyed AZ91 exhibited a higher surface roughness than AZ31 with the same preparation parameters [30]. Therefore, further work regarding the effect of alloying elements on porosity needs to be conducted to understand this phenomenon fully.

The formation of the PEO layer clearly had a significant impact on the corrosion resistance of the investigated materials. After 2 h of immersion in the corrosive media, the overall corrosion resistance manifested by the polarization resistance R_sum_ in all samples exhibited a significant increase. The only exception was the Z1-5 min sample, which exhibited the highest porosity of the coating of all 5 min samples. Therefore, the combination of relatively thin and highly porous layers did not positively impact the corrosion resistance, and faster local degradation occurred. Otherwise, the highest corrosion resistance was measured for the WE43-15 min sample, which showed the best combination of high PEO layer thickness and low porosity.

**Figure 9:**
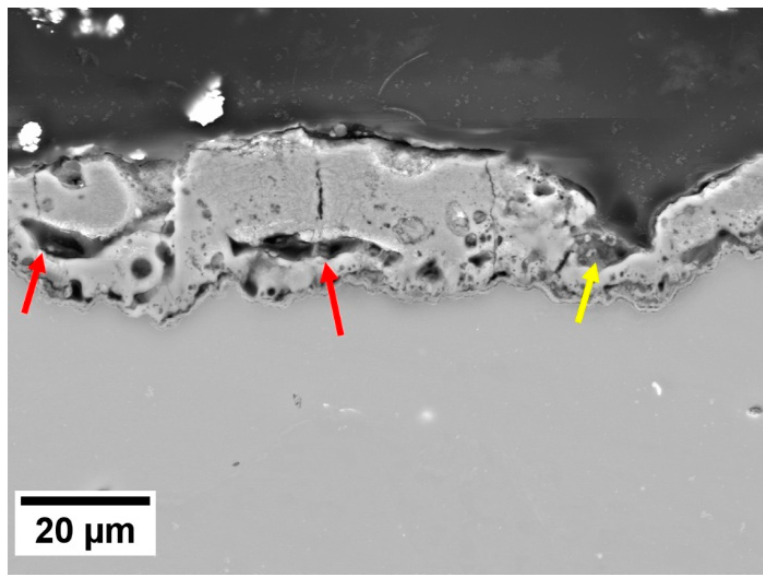
Cross section of the PEO layer formed on the ZX10-10 min sample with highlighted pores.

The PEO layer is commonly described to be composed of three parts—the outer porous layer, the intermediate dense layer, and the inner dense layer [26]. The intermediate dense layer is responsible for the good corrosion resistance of the material, and the inner dense layer for a good attachment to the surface. The cross-section analysis showed that the inner dense layer was not compact through the whole surface of any of the studied samples and often contained pores encapsulated in the layer (red arrows in Figure 9) and columnar pores reaching the inner dense layer (yellow arrows in Figure 9). The rapid decrease in R_sum_ within the first 24 h of immersion observed for all investigated samples can be associated with the dissolution of the inner dense layer at the bottom of the pores and the onset of degradation of the substrate. The further increase in the immersion time resulted in a continuous increase in the corrosion resistance in most of the samples because of the formation of a corrosion layer below the PEO layer on the coating–substrate interface (cf. Figure 7 and Figure 8). The formation of primarily Mg(OH)_2_ hydroxide [31] occurred at the beginning in the points of PEO layer failure. These products tend to fill the faults and defects of the PEO coating, thereby locally improving its integrity and also increasing the barrier effect of the coating [24]. Subsequently, a uniform layer of the corrosion products formed on the whole surface together with a continuous dissolution of the PEO layer, which became significantly thinner and highly fragmented (Figure 8). As a result of the PEO layer dissolution, the phosphates released from the dissolved PEO layer partially built into the newly created corrosion layer in the case of all samples (see Figure 8), making it more compact and protective. Phosphates are well known for inhibiting the corrosion process in biological media, which are rich in these elements [32]. The results showed that the most convenient PEO preparation time for all studied materials was 10 min (Figure 7). This result is in good agreement with the literature, where it has been repeatedly shown that the barrier effect of the PEO layer may be damaged by prolonged preparation time due to the formation of higher porosity and crack density [20]. The same effect was observed also in this study (cf. Figure 3 and Figure 6).

Finally, the investigated alloys Z1 and ZX10 showed slightly lower average corrosion resistance after 168 h of immersion in 0.9% NaCl solution than the reference ones (Mg and WE43). Nevertheless, note that the corrosion resistance of all investigated alloys was comparable after 168 h of immersion, considering the statistical error. This result is very important regarding the development of biodegradable magnesium-based materials because it shows that the low-alloyed alloys with high biocompatibility, like Mg-Zn(-Ca), can achieve comparable corrosion resistance to a highly corrosion-resistant WE43 alloy by a proper surface treatment. This result is of particular interest because of the recent successful in vivo test of WE43-based magnesium implants with PEO surface modification [8]. In addition, it is shown that the formation of the PEO layer is not beneficial only before its dissolution, but a transfer of phosphate into the newly created magnesium hydroxide improves the corrosion resistance of the material, even for a longer time.

## 5. Conclusions

The subject of this study was the analysis of the influence of the preparation time of the PEO process on the degradation resistance of Mg-1Zn (Z1) and Mg-1Zn-0.4Ca (ZX10) alloys in comparison to the pure Mg and commercial Mg-4Y-3RE-0.4Zr (WE43) prepared similarly. The following conclusions may be drawn from this study.

- The morphology of the PEO layers showed a typical pore-like nature. A longer preparation time caused an increase in the size and coverage of pores on the surface of all materials, as well as a growth in microcracks’ size and density. The cross-section observation showed that the PEO layers prepared for 10 min were the most homogeneous ones.
- The corrosion resistance measured by EIS showed that the best results were achieved after 10 min of PEO preparation. Overall, the corrosion resistance of all investigated materials was higher than that of their PEO-free counterparts, as shown in the results measured after two hours of immersion.
- The corrosion resistance of all investigated alloys was comparable within the statistical error after 168 h of immersion. This result shows that the low-alloyed alloys with high biocompatibility, like Mg-Zn(-Ca), can achieve comparable long-term corrosion resistance to the highly corrosion-resistant WE43 alloy by proper surface treatment.
- The cross-sectional and chemical analysis of the samples after 168 h of immersion showed that the PEO layer degrades during the immersion and the corrosion layer develops beneath it. The dissolved phosphates build into the newly formed corrosion layer and improve its properties. The formation of the PEO layer is not beneficial only before its dissolution, but a transfer of phosphate into the newly created magnesium hydroxide improves the corrosion resistance of the material, even for a longer time.

## Figures and Tables

**Table 1:** Average thickness of the PEO layers *d* evaluated from cross sections in the pore-free areas.

	d (µm)			
	Z1	ZX10	Mg	WE43
**5 min**	12 ± 1	13 ± 1	12 ± 1	7 ± 1
**10 min**	18 ± 2	14 ± 1	17 ± 2	14 ± 1
**15 min**	25 ± 2	23 ± 2	22 ± 2	27 ± 2

**Table 2:** Average EDS composition of PEO layer formed on the 10 min samples excluding oxygen and phosphorous.

	Mg	Zn	Ca	Y	Nd
**Z1**	99.6 ± 0.1	0.4 ± 0.1	-	-	-
**ZX10**	98.8 ± 0.1	0.3 ± 0.1	1.0 ± 0.1	-	-
**WE43**	93.7 ± 0.1	-	-	3.3 ± 0.1	3.0 ± 0.1

## Data Availability

The original contributions presented in the study are included in the article, further inquiries can be directed to the corresponding author.
